# A Path to Better Nursing: Fostering Person-Centered, Proactive Nursing Care

**DOI:** 10.1097/jnr.0000000000000415

**Published:** 2020-11-12

**Authors:** Shiow-Ching SHUN

We nurses always set the bar high for the nursing care that we provide to our patients. Although ‘better nursing’ reflects the pursuit of ever higher goals in the nursing profession, the underlying concept varies by time and situation. This is especially true in light of our aging society, the increasing role of artificial intelligence in healthcare (Risling & Low, 2019), and the strong current influence of COVID-19. These and other challenges make defining and assessing “good nurses” and “better nursing” a particularly difficult challenge for nursing today. As a result, a well-designed tool to assess better nursing may offer good indicators for how to improve care quality and achieve better nursing. In our aging society, we face a rise in chronic-disease issues among elderly living in nursing home and community settings. Hence, how to empower our patients as well as their caregivers through teaching techniques for better self-care is crucial to achieving better nursing (Schoberer et al., 2016).

This issue of *The Journal of Nursing Research* presents several studies related to older-adult populations and chronic disease self-care (e. g., diabetes and coronary artery diseases). Importantly, Park and Park (2020) developed the Good Nurse Questionnaire (17 items) and Better Nursing Questionnaire (16 items) as assessments for ‘good nurses’ and ‘better nursing’. As the authors note, these measures may be used in different country and cultural settings to assess potential differences in these concepts. Another study in this issue examined the influence of intrinsic motivation on pay satisfaction among caregivers employed in residential homes, finding that the caregivers with higher levels of intrinsic motivation reported higher pay satisfaction and lower job burnout. In nursing education, fostering the intrinsic motivation of nursing students may be an effective strategy for cultivating good nurses and better nursing practices. Proactive nursing is rooted in intrinsic motivation.

Issues related to older adult populations and nursing homes are increasing, and half of the studies in this issue focus on these issues. Informal caregivers play significant roles in caring for older adults at home, and their caring competence affects their caregiving quality. In one study, better caregiver competence was found to improve the quality of the home-based care available to older adults with disabilities. Person-centered nursing that is rooted in informal caregiver training with care and rehabilitation knowledge and skills to improve the competencies of caregivers is suggested. Another study assessed the ego-integrity management of older adults by nurses in nursing home environments. Frailty is one of the manifestations of geriatric syndrome and is a major concern in aging societies. Li et al. found the prefrail period to be an important predictor of health-related quality of life, and may be a valuable indicator that helps nurses better prevent frailty in their patients. Better self-care provides a rule of thumb to improve quality of life among patients with chronic disease. The key issue is how to educate or train these patients to improve their self-care capabilities. One study applied Orem’s theory to try to improve self-care abilities and quality of life in patients with coronary artery disease, finding that the training program was effective in improving quality of life.

This issue offers good evidence regarding the positive effects of person-centered nursing, proactive nursing, and expertise on caring for older-adult populations and patients with chronic disease. I hope this issue helps you along the road to becoming an even better nurse.
